# Mometasone Furoate Inhibits the Progression of Head and Neck Squamous Cell Carcinoma via Regulating Protein Tyrosine Phosphatase Non-Receptor Type 11

**DOI:** 10.3390/biomedicines11102597

**Published:** 2023-09-22

**Authors:** Lin Qiu, Qian Gao, Anqi Tao, Jiuhui Jiang, Cuiying Li

**Affiliations:** 1 Central Laboratory, Peking University School and Hospital of Stomatology & National Center for Stomatology & National Clinical Research Center for Oral Diseases & National Engineering Research Center of Oral Biomaterials and Digital Medical Devices, Beijing 100081, Chinagaoqian1995@bjmu.edu.cn (Q.G.); 2011110515@stu.pku.edu.cn (A.T.); 2 Department of Orthodontics, Peking University School and Hospital of Stomatology & National Center for Stomatology & National Clinical Research Center for Oral Diseases & National Engineering Research Center of Oral Biomaterials and Digital Medical Devices, Beijing 100081, China

**Keywords:** head and neck squamous cell carcinoma, mometasone furoate, proliferation, apoptosis, network pharmacology, PTPN11

## Abstract

Mometasone furoate (MF) is a kind of glucocorticoid with extensive pharmacological actions, including inhibiting tumor progression; however, the role of MF in head and neck squamous cell carcinoma (HNSCC) is still unclear. This study aimed to evaluate the inhibitory effect of MF against HNSCC and investigate its underlying mechanisms. Cell viability, colony formation, cell cycle and cell apoptosis were analyzed to explore the effect of MF on HNSCC cells. A xenograft study model was used to investigate the effect of MF on HNSCC in vivo. The core targets of MF for HNSCC were identified using network pharmacology analysis, TCGA database analysis and real-time PCR. Molecular docking was performed to determine the binding energy. Protein tyrosine phosphatase non-receptor type 11 (PTPN11)-overexpressing cells were constructed, and then, the cell viability and the expression levels of proliferation- and apoptosis-related proteins were detected after treatment with MF to explore the role of PTPN11 in the inhibitory effect of MF against HNSCC. After cells were treated with MF, cell viability and the number of colonies were decreased, the cell cycle was arrested and cell apoptosis was increased. The xenograft study results showed that MF could inhibit cell proliferation via promoting cell apoptosis in vivo. PTPN11 was shown to be the core target of MF against HNSCC via network pharmacology analysis, TCGA database analysis and real-time PCR. The molecular docking results revealed that PTPN11 exhibited the strongest ability to bind to MF. Finally, MF could attenuate the effects of increased cell viability and decreased cell apoptosis caused by PTPN11 overexpression, suggesting that MF can inhibit the progression of HNSCC by regulating PTPN11. MF targeted PTPN11, promoting cell cycle arrest and cell apoptosis, and consequently exerting effective anti-tumor activity.

## 1. Introduction

Head and neck squamous cell carcinoma (HNSCC) is the sixth most common form of malignant tumor in the world. The incidence rate is increasing and is expected to increase by 30% by 2030 [1]. The traditional treatment for HNSCC includes surgery followed by chemoradiotherapy. Despite the multimodal treatment strategy, over half of patients experience relapse or metastasis. For patients with recurrent/metastatic HNSCC, combined chemotherapy with platinum and paclitaxel or 5-fluorouracil plus the EGFR monoclonal antibody cetuximab is the standard first-line therapeutic regimen, but consequential drug resistance is common and finally leads to the limited efficacy of treatment [2]. The FDA-approved immune checkpoint inhibitor pembrolizumab is an antibody to PD1 and can effectively improve the survival rate of recurrent/metastatic HNSCC patients, although only the patients who express PD-L1 can benefit from immune checkpoint inhibitor therapy. Meanwhile, the serious adverse reactions caused by immune checkpoint inhibitor therapies need to be taken into consideration for administration. To date, the median survival of recurrent/metastatic HNSCC patients is only 11.6 months [3]. Therefore, drug treatment methods that are more effective and present fewer side effects are still urgently required.

Glucocorticoids are a class of steroidal hormones that bind to the glucocorticoid receptor to become involved in the regulation of multiple key biological processes, including inflammation and glucose metabolism [4]. While classically used as an anti-inflammation drug, recently accumulating evidence demonstrates that glucocorticoids can also treat malignant tumors. It is well established that glucocorticoids are the cornerstone of lymphatic cancer treatment due to their verified functions of arresting cell growth and promoting apoptosis [5,6]. Most importantly, the activation of cell cycle arrest and apoptosis are common ways for drugs to perform anti-cancer activity in multiple cancers, including HNSCC [7,8]. Mometasone furoate (MF) is a kind of glucocorticoid with extensive pharmacological action. Previous studies regarding MF have mostly focused on its use as a treatment for inflammation. In a recent study, MF inhibited the growth and induced the apoptosis of acute leukemia cells by regulating the PI3K signaling pathway [9]. For the above-mentioned reasons, MF was identified as a promising anti-cancer drug for HNSCC. Nevertheless, the inhibitory role and underlying mechanisms of MF against HNSCC remain to be determined.

Network pharmacology is a comparatively comprehensive and systematic way to predict the potential targets of clinical drugs [10,11]. Molecular docking is an important method to predict the binding between targets and drugs. The combination of these two methods provides a better reference for the application of clinical drugs and the repurposing of precise and effective therapeutic drugs. Therefore, it is an effective adjuvant method to screen the targets and underlying mechanisms of MF against HNSCC.

Protein tyrosine phosphatase non-receptor type 11 (PTPN11) is a member of the protein tyrosine phosphatase (PTP) family and is the first proto-oncogene receptor tyrosine phosphatase. PTPs work in coordination with protein tyrosine kinases (PTKs) to balance the phosphorylation status of tyrosine in signaling proteins, which determines multiple cellular processes through the transduction of signaling cascades. PTPN11 is required by most receptor tyrosine kinases (RTKs) to activate the downstream signaling pathways. As a result, PTPN11 plays a central role in the activation of oncogenic signaling pathways, such as PI3K/AKT [12], RAS/Raf/MAPK [13] and Jak/STAT [14]. It is widely acknowledged that PTPN11 is highly expressed in many tumors [15], and its aberrant expression is closely related to a poorer prognosis in a range of tumors [16,17]. In HNSCC, PTPN11 is overexpressed and participates in the invasion and metastasis of cells via activating the ERK1/2-Snail/Twist1 pathway [18]. Overexpressed PTPN11 could contribute to antigen-processing-machinery-component downregulation, thus leading to cytotoxic T lymphocyte evasion [19]. Accordingly, PTPN11 could be regarded as an effective target for tumor therapy in HNSCC [20].

## 2. Materials and Methods

### 2.1. Cell Culture

Cells from two human HNSCC cell lines, WSU-HN6 and CAL-27, were purchased from the American Type Culture Collection. Both cells were cultured in high-glucose DMEM (Gibco, New York, NY, USA) with 10% FBS (Gibco, New York, NY, USA) and 1% penicillin/streptomycin solution (Solarbio, Beijing, China) and maintained in 5% CO_2_ at 37 °C.

### 2.2. Cell Counting Kit-8 Assay

The cell viability was measured using CCK-8 (Beyotime, Shanghai, China) according to the manufacturer’s protocol. Cells were seeded into 96-well plates at a density of 4000 cells/well. After 24, 48 and 72 h, the supernatant was replaced by serum-free DMEM and CCK-8 solution (10:1) and incubated for 2 h at 37 °C. The optical density (OD) at 450 nm was measured using an automatic microplate reader (BioTek ELX808, Biotek Instruments, Vermont, VT, USA).

Cell viability was calculated using the formula: cell viability = [(experimental wells’ OD − blank wells’ OD)/(control wells’ OD − blank wells’ OD)] × 100%); OD450 = experimental wells’ OD − blank wells’ OD.

### 2.3. Colony Formation Assay

Cells were plated into 6-well plates at a density of 400 cells/well and cultured for 7 days. The culture medium was changed every two days. When the cell clone was visible to the naked eye, it was washed with phosphate-buffered saline (PBS), fixed with formaldehyde for 15 min and stained with 0.5% crystal violet at room temperature for 20 min. Finally, the cell clones were rinsed with PBS and the photographs were captured via a scanner (HP Scanjet G4050, China Hewlett-Packard Co., Ltd., Beijing, China).

### 2.4. Flow Cytometric Analysis

Cells were seeded into 6-well plates at 3 × 10^5^ cells/well and cultured for 48 h. For the analysis of the cell cycle, the cells were fixed in 70% ethanol overnight at 4 °C, rinsed twice with PBS and stained with 500 μL of buffer, 25 μL of PI and 10 μL of RNase A at 37 °C for 30 min using the Cell Cycle and Apoptosis Analysis Kit (Beyotime, Shanghai, China) according to the manufacturer’s instructions. For the analysis of apoptosis, the cells were rinsed twice with PBS and were stained with 5 μL of Annexin V-FITC and 5 μL of PI for 15 min using the Annexin V-FITC Apoptosis Detection Kit (Solarbio, Beijing, China) according to the manufacturer’s instructions. Then, the DNA content and apoptosis rate of cells were examined via flow cytometry (Beckman, Brea, CA, USA).

### 2.5. Western Blotting

The Western blotting protocol was based on our earlier publication [21]. Briefly, collected tissues and cells were lysed using RIPA buffer (Solarbio, Beijing, China) complemented with 100 mM PMSF on ice. The BCA Protein Assay Kit (Beyotime, Shanghai, China) was employed to detect the concentrations of proteins. A total of 20 μg of protein from each sample was added to each lane of a 4–12% SDS polyacrylamide gel to run SDS-PAGE, and then the proteins were transferred to PVDF membranes and blocked with 5% BSA for 1 h at room temperature. The membranes were incubated with primary antibodies overnight at 4 °C and the corresponding secondary antibodies for 1 h at room temperature. The antibodies used included ki67 (1:1000, Beyotime, Shanghai, China), PCNA (1:1000, Beyotime, Shanghai, China), cleaved caspase-3 (1:1000, CST, Danvers, MA, USA), Bax (1:1000, Wanleibio, Shenyang,, China), Bcl-2 (1:1000, Wanleibio, Shenyang, China), GAPDH (1:10,000, Proteintech, Wuhan, China), PTPN11 (1:1000, Wanleibio, Shenyang, China) and HRP-labeled goat anti-rabbit IgG (1:1000, Beyotime, Shanghai, China).

### 2.6. Real-Time PCR Analysis

Total RNA was extracted using Trizol (Invitrogen, Carlsbad, CA, USA), and then the equivalent RNA of each group was reverse-transcribed into cDNA using a Prime ScriptTM RT reagent Kit (TaKaRa, Gunma, Japan). Real-time PCR reactions were conducted in 10 μL of mixture including 1 μL of each cDNA sample, 0.5 μL of specific forward primers (10 μM), 0.5 μL of specific reverse primers (10 μM), 5 μL of 2X Universal SYBR Green Fast qPCR Mix (ABclonal, Wuhan, China) and 3 μL of double-distilled water. The conditions were 95 °C for 5 min followed by 40 cycles of 95 °C for 15 s, 60 °C for 30 s and 72 °C for 30 s, and finally 72 °C for 10 min. All data were normalized to GAPDH. The primer sequences are listed in Table 1.

### 2.7. Xenograft Study Models

All animal experiments were approved by the Institutional Animal Care and Use Committee of the Peking University Health Science Center (No. LA2022229), following the Committee of Peking University Health Science Center’s Animals Usage Guidelines and performed using the approved protocols of the Animal Ethical and Welfare Committee. Healthy male BALB/cA-nu mice (4–5 weeks) were injected with 5 × 10^6^ cells in 100 μL of PBS subcutaneously (two groups were injected with WSU-HN6 cells, and the other two groups were injected with CAL-27 cells, four mice/group). When the tumor volume was about 100 mm^3^, 15 mg/kg MF was administered to mice twice per week orally by gavage and an equal volume of DMSO alone (0 mg/kg MF) was given to the control group. Tumor size was measured every 3 days and estimated using the formula: V = length × width^2^/2. After 4 weeks, the mice were euthanized and the tumors were removed entirely to conduct HE and Western blotting.

### 2.8. Hematoxylin–Eosin Staining

Hematoxylin–eosin (HE) staining was performed to determine whether organ toxicity occurred due to the MF treatment in vivo. The corresponding tissues from mice were collected and fixed in 4% paraformaldehyde, embedded in paraffin wax and 5 μm sections were cut and mounted onto slides. The slides were deparaffinized, rehydrated and stained with hematoxylin and eosin. Then, the images were captured using a fluorescence microscope (Nikon, Minato-ku, Japan).

### 2.9. Bioinformatics Analysis

Pharmmapper (http://www.lilab-ecust.cn/pharmmapper/, accessed on 13 April 2023) was employed to screen for potential targets of MF. HNSCC-related genes were obtained from GeneCards (https://www.genecards.org/, accessed on 13 April 2023) and the Comparative Toxicogenomics Database (CTD, http://ctdbase.org/, accessed on 13 April 2023). The intersection of potential MF targets and HNSCC-related genes was set as the potential targets of MF in HNSCC; these genes were imported into STRING (https://string-db.org/, accessed on 14 April 2023) to construct a PPI network, and then degree centrality (DC), closeness centrality (CC), eigenvector centrality (EC), betweenness centrality (BC), local average connectivity (LAC) and network centrality (NC) were used to identify the core targets of MF against HNSCC via Cytoscape 3.7.1.

To further investigate the underlying mechanisms of MF against HNSCC, the potential targets were used to perform Gene Ontology (GO) and Kyoto Encyclopedia of Genes and Genomes (KEGG) pathway analysis using the Database for Annotation Visualization and Integrated Discovery (DAVID, https://david.ncifcrf.gov/, accessed on 15 April 2023).

To valid the results of the network pharmacology analysis, the RNA sequencing (FPKM) and clinical information about HNSCC were accessed before August 2022 from The Cancer Genome Atlas (TCGA) database (https://portal.gdc.cancer.gov/, accessed on 29 August 2023). Then, the expression levels of MF core targets were detected and survival analysis was performed.

### 2.10. Molecular Docking

**Figure 1:**
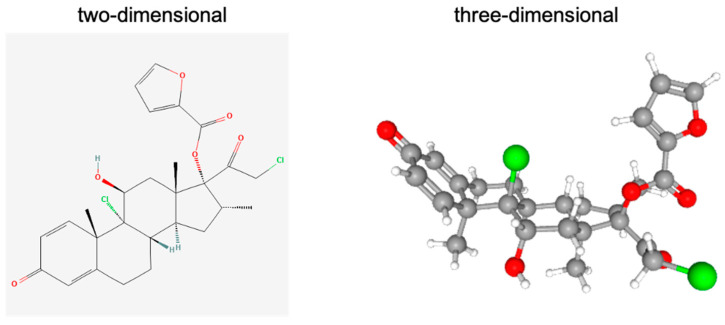
The structure of MF (image downloaded from PubChemd, Compound CID: 441336).

The structure of MF was downloaded from PubChem (https://pubchem.ncbi.nlm.nih.gov/, Compound CID: 441336, accessed on 13 April 2023) and is shown in Figure 1. The protein structures were obtained from PDB (http://www.rcsb.org/, PDB ID: EGFR: 1m14, GRB2: 1bm2, IGF1R: 1igr, SRC: 1a07, PTPN11: 2shp, MAPK1: 1pme, accessed on 15 April 2023). PyMOL software (version 4.6.0) was used to remove the water and small-molecule ligands of the protein. AutoDockTools (version 1.5.6) was employed to hydrotreat the protein molecules, obtain PDBQT files and determine active pockets. We ran the Vina 1.1.2 program to calculate the binding energy. Finally, the optimal combination model was visualized via PyMOL. A smaller binding energy indicated a stronger binding force between MF and the target proteins. A binding energy ≤ −5.0 kcal/mol indicated they could be combined, and a binding energy ≤ −7.0 kcal/mol indicated that they exhibited excellent binding strength.

### 2.11. Construction of PTPN11-Overexpression Plasmid and Cell Lines

To explore whether PTPN11 acts as a downstream target of MF, we constructed stable PTPN11-overexpressing WSU-HN6 and CAL-27 cells. The PCDH plasmid was employed as the control group (scramble cells). Human full-length PTPN11 cDNA was amplified and cloned into the PCDH plasmid using the ClonExpress II One Step Cloning Kit (Vazyme, Nanjing, China) according to the manufacturer’s protocol. The primer is shown in Table 2. The plasmid with the correct sequence was transfected into 293T cells with VSVG and PAX8 plasmids. Lentivirus supernatants were harvested at 48 h and were utilized to infect WSU-HN6 and CAL-27 cells at 80% confluency. Puromycin was added 48 h after infection to obtain the positive PTPN11-overexpressed WSU-HN6 and CAL-27 cells. Real-time PCR and Western blotting were performed to detect the overexpression efficiency at the RNA level and protein level, respectively, according to the methods described detailed in the corresponding sections. Additionally, the stable PTPN11-overexpressing cells were treated with MF, and then cell viability and Western blotting were conducted to detect the role of PTPN11 in the anti-tumor efficacy of MF.

### 2.12. Statistical Analysis

GraphPad Prism 8.4.3 software (San Diego, CA, USA) was employed for the statistical analyses. All experiments were repeated three times to ensure the validity of the data. All data are expressed as the mean ± standard deviation (SD, *n* = 3). Data for more than two groups were tested for homogeneity, followed by one-way ANOVA analysis. *p* < 0.05 was considered statistically significant.

## 3. Results

### 3.1. MF Inhibited the Proliferation of HNSCC Cells

**Figure 2:**
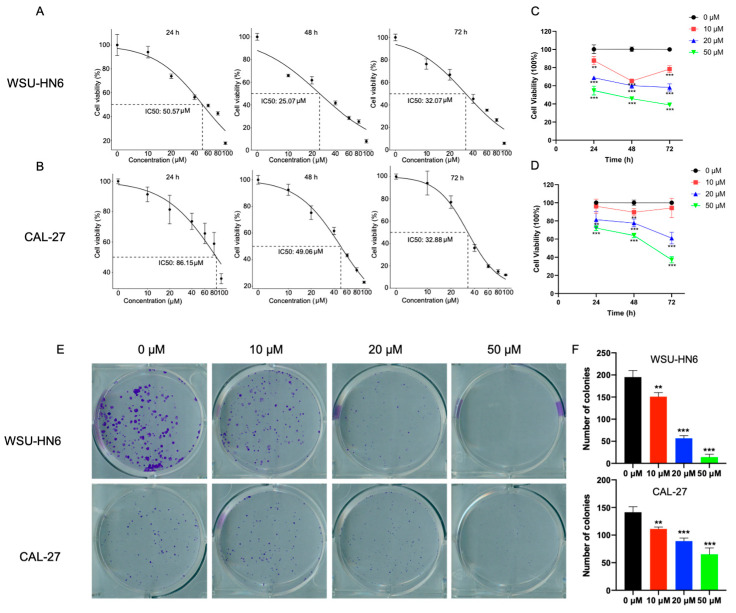
The effect of MF on the cell proliferation of HNSCC cells in vitro. (**A**,**B**) The IC_50_ of WSU-HN6 cells (**A**) and CAL-27 cells (**B**) were detected after cells were treated with 0, 10, 20, 40, 60, 80 and 100 μM MF for 24, 48 and 72 h; (**C**,**D**) the percentage of viability WSU-HN6 cells (**C**) and CAL-27 cells (**D**) after cells were treated with 0, 10, 20 and 50 μM MF for 24, 48 and 72 h; (**E**) the colony formation of WSU-HN6 and CAL-27 cells after cells were treated with 0, 10, 20 and 50 μM MF for 7 days; (**F**) the quantitative analysis results of (**E**). ** *p* < 0.01, *** *p* < 0.001, *n* = 3. Asterisks represent differences between cells treated with 10, 20 and 50 μM MF and cells treated with 0 μM MF.

To determine the cell cytotoxicity of MF, we first detected the half-maximal inhibitory concentration (IC_50_) of cells treated with MF, which decreased as time increased in CAL-27 cells (Figure 2B). The IC_50_ of WSU-HN6 cells treated with MF was decreased after 48 h of culture compared with 24 h of culture (24 h: 50.57 μM; 48 h: 25.07 μM). Meanwhile, after 72 h of culture, the IC_50_ of WSU-HN6 (72 h: 32.07 μM) was slightly higher than that of 48 h of culture (Figure 2A). According to the IC_50_, we chose 0, 10, 20 and 50 μM for further experiments. Then, cell viability was detected after cells were treated with different doses of MF and incubated for different periods of time (24, 48 and 72 h). Cell viability gradually decreased as the dose and time increased (Figure 2C,D), indicating that MF could inhibit cell proliferation in a time- and dose-dependent manner. Similar results were observed in the colony formation assay. The number of colonies in the MF-treated groups decreased with increasing concentrations of MF (Figure 2E,F). Accordingly, these data recapitulated the inhibitory effect of MF on the proliferation of HNSCC cells.

### 3.2. MF Regulated the Cell Cycle and Induced Apoptosis In Vitro

**Figure 3:**
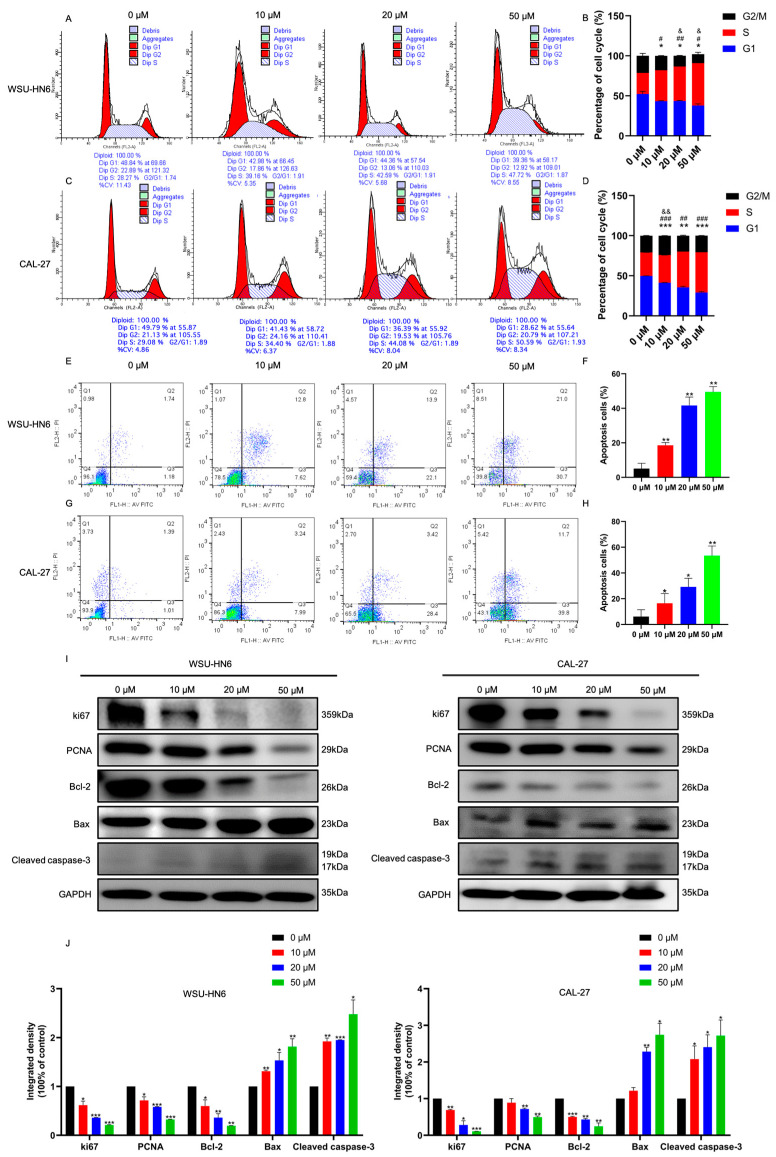
MF induced cell cycle arrest and cell apoptosis of HNSCC cells in vitro. (**A**–**D**) Distribution (**A**,**C**) and statistical analysis (**B**,**D**) of cell cycles of WSU-HN6 and CAL-27 cells, respectively, treated with 0, 10, 20 and 50 μM MF for 24 h; (**E**–**H**) representative flow cytometry plots (**E**,**G**) and statistical analysis of the cell apoptosis rate (**F**,**H**) of WSU-HN6 and CAL-27 cells, respectively, treated with 0, 10, 20 and 50 μM MF for 24 h; (**I**) the expression levels of proliferation- and apoptosis-related proteins in WSU-HN6 and CAL-27 cells treated with 0, 10, 20 and 50 μM MF; (**J**) the quantitative analysis results of (**I**). *, #, & *p* < 0.05; **, ##, && *p* < 0.01; ***, ### *p* < 0.001; *n* = 3. Asterisks represents the difference between G1 phase, apoptosis or the corresponding protein expression levels of cells treated with 10, 20 and 50 μM MF (MF-treated groups) and cells treated with 0 μM MF (control group), pounds represent the difference between S phase in MF-treated groups and control group; ampersands represents the difference between G2/M phase in MF-treated groups and control group.

Since glucocorticoids suppress lymphoid progression via regulating the cell cycle and apoptosis, we studied the cell cycle and apoptosis of cells treated with MF further to obtain insight into the proliferation inhibition effect of MF. Compared with the control group, cells treated with MF performed S-phase arrest (Figure 3A–D). Meanwhile, the cell apoptosis rate increased significantly elevated with increasing concentrations of MF (Figure 3E–H). This notion was further supported by the observation that the protein expression levels of the cell proliferation markers ki67, PCNA and anti-apoptotic protein Bcl-2 decreased and the apoptosis-related proteins Bax and cleaved caspase-3 increased with increasing concentrations of MF (Figure 3I,J). Collectively, these results demonstrated that MF inhibited cell proliferation via inducing cell cycle arrest and cell apoptosis.

### 3.3. MF Suppressed Tumor Growth In Vivo

**Figure 4:**
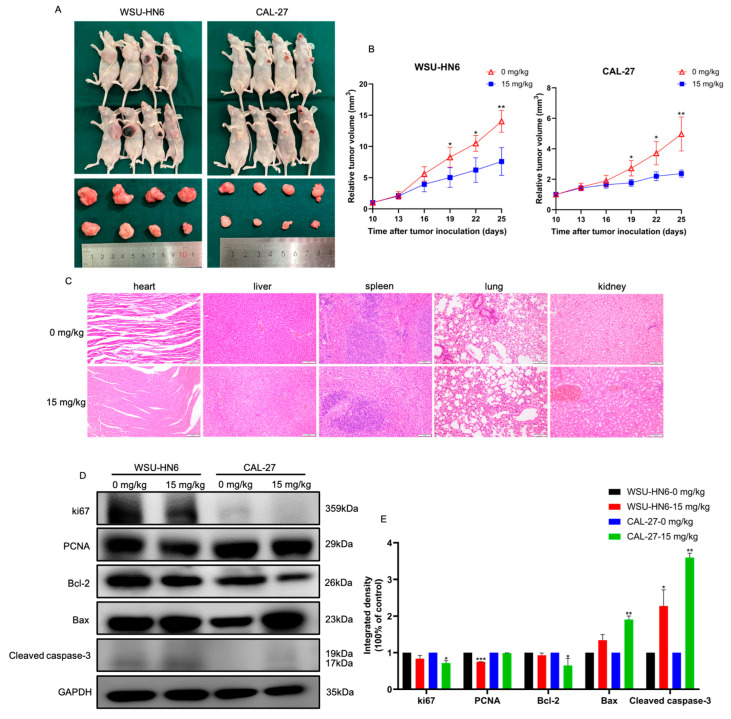
MF inhibited HNSCC progression in vivo. (**A**) Representative images of tumor-bearing mice and tumor samples; (**B**) tumor volume of the mice treated with 15 mg/kg MF compared with the mice treated with 0 mg/kg MF; (**C**) HE staining images of heart, liver, spleen, lung and kidneys of the mice treated with 0 mg/kg and 15 mg/kg MF, 20×; (**D**) the expression levels of proliferation- and apoptosis-related proteins of the mice treated with 0 mg/kg and 15 mg/kg MF; (**E**) the quantitative analysis results of (**D**). * *p* < 0.05, ** *p* < 0.01, *** *p* < 0.001, *n* = 3. Asterisks represents the difference between mice treated with 15 mg/kg MF and mice treated with 0 mg/kg MF.

We then examined whether MF could inhibit cell proliferation in vivo. Nude mice were injected with HNSCC cells and then orally administered MF at a concentration of 15 mg/kg when the tumor volume was about 100 mm^3^ (the tenth day after cell injection). Mice given an equal volume of DMSO (0 mg/kg MF) were employed as the control group. Compared with the control group, the tumor volume in the MF-treated group was remarkably decreased after 19 days of tumor inoculation, indicating that MF suppressed HNSCC cell growth in vivo (Figure 4A,B). There was no organ toxicity in the MF-treated group relative to the control group (Figure 4C). Western blotting revealed the downregulated protein expression levels of ki67, PCNA and Bcl-2 and the upregulated protein expression levels of Bax and Cleaved caspase-3 in tumors of mice treated with MF (Figure 4D,E).

### 3.4. PTPN11 Was the Core Target of MF against HNSCC

**Figure 5:**
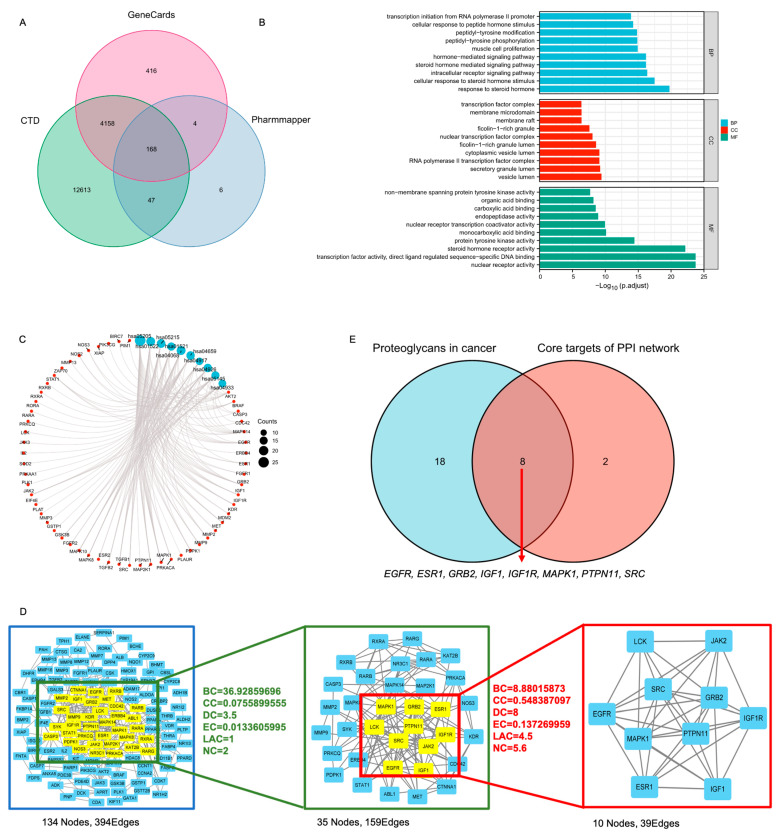
Network pharmacology analysis of the MF targets against HNSCC. (**A**) Venn diagram of MF targets and HNSCC-related genes; (**B**) chart of the top ten GO enrichments; (**C**) chart of the top ten KEGG pathways; (**D**) the PPI network constructed by Cytoscape via two-step topology analysis. The parameters of the first step are shown in green text and the parameters of the second step are shown in red text; (**E**) Venn diagram of 10 targets of MF against HNSCC and 26 genes enriched with proteoglycans in cancer.

**Figure 6:**
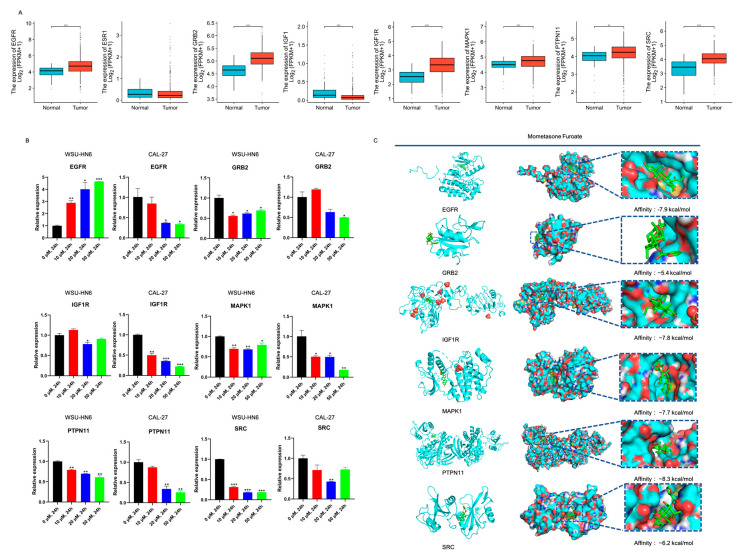
PTPN11 was the core target of MF against HNSCC. (**A**) The expression of the eight targets in TCGA database. The blue boxes represent normal tissue (*n* = 44) and the red boxes represent HNSCC tumor tissue (*n* = 504); (**B**) the mRNA expression levels of the six targets of MF against HNSCC after cells were treated with 0, 10, 20 and 50 μM MF for 24 h; (**C**) molecular docking of the six targets of MF against HNSCC. The affinity represents the binding energy. * *p* < 0.05, ** *p* < 0.01, *** *p* < 0.001, *n* = 3. Asterisks represents the differences between HNSCC tumor tissues and normal tissue or cells treated with 10, 20 and 50 μM MF and cells treated with 0 μM MF.

We then performed network pharmacology analysis to identify the potential targets of MF against HNSCC. A total of 168 genes were set as the potential targets of MF against HNSCC which were obtained from the intersection of 225 genes of MF targets from PharmMapper, and 4748 genes and 16,986 genes associated with HNSCC from GeneCards and CTD, respectively (Figure 5A). The GO analysis showed that the 168 genes were mainly associated with the response to steroid hormone and nuclear receptor activity (Figure 5B). KEGG analysis revealed that proteoglycans in cancer was the most significant enrichment signaling pathway for the potential targets of MF against HNSCC (Figure 5C, Table 3). Overall, 168 genes were imported into the STRING database and then a primary PPI network with 394 edges and 134 nodes (namely targets) was obtained; the 134 targets were examined with a two-step topology analysis and finally 10 targets were identified (Figure 5D). To further narrow the potential functional targets, we analyzed the intersection of the 10 targets and 26 genes enriched with proteoglycans in cancer. As a result, eight genes, *EGFR, ESR1, GRB2, IGF1, IGF1R, MAPK1, PTPN11* and *SRC*, were identified (Figure 5E). *ESR1* and *IGF1* were ruled out due to their non-significant and downregulated mRNA expression levels in HNSCC compared with normal tissues after analyzing the TCGA database (Figure 6A). We also analyzed the relationship between the expression levels of these potential targets and patient survival, and found that only the expression levels of *EGFR*, *IGF1R*, *PTPN11* and *ESR1* were significantly associated with patient prognosis (Supplementary Figure S1). We then detected the mRNA expression levels of six genes after cells were treated with MF. Interestingly, only the expression level of *PTPN11* notably decreased in a MF dose-dependent manner (Figure 6B). The molecular docking analysis revealed that PTPN11 showed the strongest combination with MF and the binding energy was −8.3 kcal/mol (Figure 6C). Taken together, these data strongly indicated that MF was involved in multiple tumor-associated signaling pathways, and, most importantly, that MF may exert its anti-tumor activity by targeting PTPN11.

### 3.5. MF Exerted Anti-Tumor Activity by Targeting PTPN11

**Figure 7:**
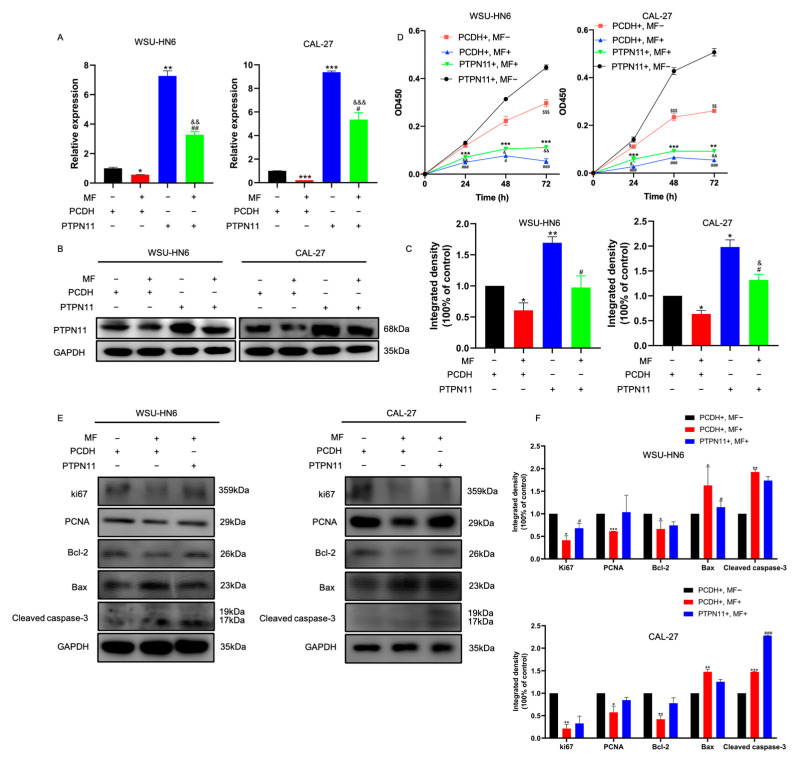
MF inhibited the HNSCC progression induced by PTPN11 overexpression. (**A**,**B**) The mRNA (**A**) and protein (**B**) expression levels of PTPN11 of the PTPN11-overexpressing cells treated with MF; (**C**) the quantitative analysis results of (**B**); (**D**) the percentage of viable PTPN11-overexpressing cells treated with and without MF; (**E**) the expression levels of proliferation- and apoptosis-related proteins of the PTPN11-overexpressing cells treated with MF; (**F**) the quantitative analysis results of (**E**). *, #, &, $ *p* < 0.05; **, ##, &&, $$ *p* < 0.01; ***, &&&, ###, $$$ *p* < 0.001; *n* = 3. Asterisks represent the difference between the PCDH+, MF+ group or the PTPN11+, MF− group and the PCDH+, MF− group (**A**,**C**). Pounds represent the difference between the PTPN11+, MF+ group and the PTPN11+, MF− group (**A**,**C**). Ampersands represent the difference between the PTPN11+, MF+ group and the PCDH+, MF+ group (**A**,**C**). Asterisks represent the difference between the PTPN11+, MF+ group cand the PTPN11+, MF− group (**D**,**F**). Pounds represent the difference between the PCDH+, MF− group and the PCDH+, MF+ group (**D**,**F**). Ampersands represents the difference between the PCDH+, MF+ group and the PTPN11+, MF+ group (**D**,**F**). Dollars represent the difference between the PTPN11+, MF− group and the PCDH+, MF− group (**D**,**F**).

To further investigate whether the inhibition of PTPN11 was required for the anti-tumor efficacy of MF, we constructed stable PTPN11-overexpressing cells. The efficiency of overexpression was confirmed at the mRNA and protein levels (Figure 7A–C). Interestingly, we observed that cells treated with MF revealed a significantly decreased expression level for PTPN11. However, there was still relatively higher expression of PTPN11 compared to the control group cells (Figure 7A–C), indicating that MF could not completely inhibit the overexpression of PTPN11. As shown in Figure 7D, the cell viability of PTPN11-overexpressing cells was significantly higher than that of scramble cells, indicating that PTPN11 could promote the proliferation of HNSCC cells, which was consistent with a previous study [22]. After cells were treated with MF, significantly decreased cell viability was observed in scramble cells and PTPN11-overexpressing cells, and the cell viability of the scramble cells was slightly lower than that of PTPN11-overexpressing cells, suggesting that PTPN11 played a central role in the inhibitory effect of MF on cell proliferation. Similar results were observed using Western blotting (Figure 7E,F): after MF treatment, the protein expression levels of ki67, PCNA and Bcl-2 in scramble cells were slightly lower than those of PTPN11-overexpressing cells. Taken together, it was shown that MF could target PTPN11 to exert anti-tumor activity.

## 4. Discussion

While they are classically used as anti-inflammation drugs, accumulating evidence has demonstrated that glucocorticoids can also treat malignant tumors such as multiple myeloma [23], prostate cancer [24], colorectal cancer [25] and breast cancer [26]. Inflammation is closely related to the development of most types of cancers. Cancer-extrinsic inflammation, triggered by elements such as viral infections and autoimmune disease, has been reported as an induced factor for tumor initiation and progression. Cancer-intrinsic inflammation is involved in the recruitment and activation of inflammatory cells, inducing an immunosuppressive tumor microenvironment and eventually accelerating malignant progression. Therefore, anti-inflammatory drugs targeting the inflammatory tumor microenvironment have been identified as the pivotal determinant for conventional chemotherapy and immunotherapy efficacy [27]. In addition, it has been reported that the overexpression of inflammation-related factors is related to drug resistance. Increased circulation of the cytokine interleukin-6 was associated with acquired resistance to dasatinib [28], which was further confirmed by a phase II clinical trial in HNSCC patients [29]. Furthermore, anti-inflammatory drugs were shown to attenuate the toxicity of chemotherapeutic agents [30]. For instance, when combined with docetaxel and celecoxib to treat patients with metastatic prostate cancer, they were shown to reduce hematologic toxicity [31]. Emerging studies have reported that local application of MF remarkably reduced acute radiation dermatitis after radiotherapy for HNSCC [32] and breast cancer [33]. Based on the aforementioned intimate relationship between inflammation and tumors, anti-inflammatory drugs seem to be a powerful method to enhance therapeutic efficiency in HNSCC.

MF is a traditional glucocorticoid with an adequate anti-inflammatory capacity. However, previous studies regarding MF have mainly focused on its use as a treatment for inflammation, and researchers have only recently begun to delineate the role of MF in cancer. Since glucocorticoids can promote cell apoptosis via binding with glucocorticoid receptors in lymphoid cells, the most extensive application of glucocorticoids in cancer is to treat lymphoid malignancies [34]. Consistent with this, it was reported that MF could inhibit the growth of acute leukemia cells through promoting cell apoptosis [9], but there was still no exploration of the pharmacological effect of MF on solid tumors. Here, we found that MF could inhibit HNSCC cell proliferation both in vitro and in vivo. Mechanistically, the protein expression level of the anti-apoptotic molecule Bcl-2 was significantly decreased and that of the pro-apoptotic Bax was increased after cells were treated with MF, which was consistent with a previous study that showed that the downregulation of Bcl-2 was essential for glucocorticoid-induced cell apoptosis [34]. Meanwhile, the decreased Bcl-2 expression subsequently released cytochrome C into the cytoplasm and then promoted the activation of cleaved caspase-3, which also is a sign of cell apoptosis. Our results also revealed that the protein expression level of cleaved caspase-3 was remarkably increased after MF treatment. These results supported the notion that MF could exert its anti-tumor effect via promoting cell apoptosis.

To better understand the inhibitory effect of MF in HNSCC, we then conducted network pharmacology and molecular docking analyses, which were effective methods to provide a more comprehensive perspective of MF’s mechanism against HNSCC. KEGG analysis demonstrated that the potential target genes of MF against HNSCC were mainly associated with proteoglycans in cancer signaling pathways, which was related to various biological processes in cancer [35]. According to a series of bioinformatics analyses, PTPN11 seemed to be the most important target of MF against HNSCC. Our experimental results confirmed that MF decreased the mRNA expression level of PTPN11 in a dose-dependent manner. It is widely known that sustained activation of PTPN11 is responsible for the occurrence, development and prognosis of multiple cancers. Our pan-cancer analysis of PTPN11 through the TCGA database also confirmed abnormal expression of PTPN11 in multiple tumors and a significant correlation with patient prognosis (Supplemental Figure S2). Allosteric inhibition of PTPN11 via SHP099 suppressed the RTK-driven human cancer cells by inhibiting the RAS-ERK signaling pathway [36]. RAS was overexpressed in HNSCC cells and was responsible for tumor growth [37]. Therefore, we assumed that MF exerted its excellent anti-tumor capacity via targeting PTPN11. To verify this, we constructed PTPN11-overexpressing cells to explore whether MF performs its anti-tumor effect by suppressing PTPN11. More interestingly, we observed that MF could decrease the mRNA and protein expression levels of PTPN11, as well as inhibit the cell proliferation and increased cell apoptosis after PTPN11 is overexpressed. However, MF could not eliminate the exogenous overexpression of PTPN11 and the promotion of cell growth caused by PTPN11 overexpression. We predicted that there were eight genes targeted by MF against HNSCC via the network pharmacology analysis. Thus, MF also has the potential to bind with other targets, resulting in a competitive combination between the targets and MF, which may explain why MF could not inhibit PTPN11 completely. Further studies are needed to clarify this issue. In addition, among the eight targets, EGFR was upregulated in 80–90% of HNSCC patients and the overexpression of EGFR was associated with a poor prognosis [38]. The FDA-approved EGFR monoclonal antibody cetuximab has been combined with chemotherapy or radiotherapy as a first-line therapy for HNSCC patients, but the acquisition of EGRF resistance was a frequent occurrence, eventually leading to therapy failure. Furthermore, PTPN11 inhibitors could overcome EGFR resistance in NSCLC [39]. Thus, we reasonably assumed that a single usage of MF could target PTPN11 to produce a marked anti-tumor effect. The combination of MF and PTPN11 inhibitors could overcome drug resistance to achieve a better therapeutic effect on HNSCC.

Overall, we proved that MF could exert an excellent anti-tumor effect through regulating PTPN11, which provides a theoretical basis for its clinical application. However, the detailed molecular mechanism still needs further exploration for clinical drug use.

## 5. Conclusions

We preliminarily revealed that MF plays a vital role in suppressing the proliferation of HNSCC via regulating the expression level of PTPN11. This provided a new perspective on the treatment of HNSCC with MF.

## Figures and Tables

**Table 1:** The real-time PCR primers.

Gene	Primer Sequence
*EGFR*	F: GGTGAGTGGCTTGTCTGGAA
*EGFR*	R: CCTTACGCCCTTCACTGTGT
*GBR2*	F: AAGCTACTGCAGACGACGAG
*GBR2*	R: CTTGGCTCTGGGGATTTTGC
*IGF1R*	F: AGGCTGGGGCTCTTGTTTAC
*IGF1R*	R: CCTCTCTCGAGTTCGCCTG
*SRC*	F: TTCTGCTGTTGACTGGCTGT
*SRC*	R: TGAGGATGGTCAGGTTGTGC
*PTPN11*	F: CGTCATGCGTGTTAGGAACG
*PTPN11*	R: TCTCTCCGTATTCCCCTGGA
*MAPK1*	F: TCCTTTGAGCCGTTTGGAGG
*MAPK1*	R: AGTACATACTGCCGCAGGTC

**Table 2:** Sequences of oligonucleotides used for PTPN11 overexpression.

Primer	Oligonucleotides Sequence
PTPN11-OE-F	GGGGGAGGAGGGGGATCCGGAATGACATCGCGGAGATGGT
PTPN11-OE-R	GATCCTTCGCGGCCGCGATCCTCATCTGAAACTTTTCTGC

**Table 3:** The top ten KEGG pathways.

ID	Description	Gene Ratio	*p* Value	p. Adjust	q Value
hsa05205	Proteoglycans in cancer	26/150	4.1534 × 10^−15^	1.109 × 10^−12^	5.2901 × 10^−13^
hsa01522	Endocrine resistance	18/150	1.4301 × 10^−13^	1.9092 × 10^−11^	9.1077 × 10^−12^
hsa05215	Prostate cancer	17/150	1.5834 × 10^−12^	1.4092 × 10^−10^	6.7226 × 10^−11^
hsa01521	EGFR tyrosine kinase inhibitor resistance	15/150	1.0373 × 10^−11^	6.9241 × 10^−10^	3.3031 × 10^−10^
hsa04068	FoxO signaling pathway	18/150	2.4517 × 10^−11^	1.3092 × 10^−9^	6.2455 × 10^−10^
hsa04659	Th17 cell differentiation	16/150	9.1379 × 10^−11^	4.0664 × 10^−9^	1.9398 × 10^−9^
hsa04917	Prolactin signaling pathway	13/150	3.5466 × 10^−10^	1.3528 × 10^−8^	6.4532 × 10^−9^
hsa04926	Relaxin signaling pathway	16/150	1.5842 × 10^−9^	5.2872 × 10^−8^	2.5222 × 10^−8^
hsa05145	Toxoplasmosis	15/150	1.7878 × 10^−9^	5.3037 × 10^−8^	2.5301 × 10^−8^
hsa04933	AGE-RAGE signaling pathway in diabetic complications	14/150	3.5417 × 10^−9^	9.3914 × 10^−8^	4.48 × 10^−8^

## Data Availability

The data supporting this study are available from the corresponding author upon reasonable request.

## Supplementary Materials

The following supporting information can be downloaded at: https://www.mdpi.com/article/10.3390/biomedicines11102597/s1, Figure S1: Survival curve of patients with HNSCC in TCGA database; Figure S2: Bioinformatic analysis of PTPN11 in multiple cancers in the TCGA database. (A) The expression characteristics of PTPN11 in multiple cancers. ACC: adrenocortical carcinoma, BLCA: bladder urothelial carcinoma, BRCA: breast invasive carcinoma, CESC: cervical endocervical adenocarcinoma and squamous cell carcinoma, CHOL: cholangiocarcinoma, COAD: colon adenocarcinoma, DLBC: lymphoid neoplasm diffuse large B-cell lymphoma, ESCA: esophageal carcinoma, GBM: glioblastoma multiforme, HNSC: head and neck squamous cell carcinoma, KICH: kidney chromophobe, KIRC: kidney renal clear cell carcinoma, KIRP: kidney renal papillary cell carcinoma, LAML: acute myeloid leukemia, LGG: brain lower grade glioma, LIHC: liver hepatocellular carcinoma, LUAD: lung adenocarcinoma, LUSC: lung squamous cell carcinoma, MESO: mesothelioma, OV: ovarian serous cystadenocarcinoma, PAAD: pancreatic adenocarcinoma, PCPG: pheochromocytoma and paraganglioma, PRAD: prostate adenocarcinoma, READ: rectum adenocarcinoma, SARC: sarcoma, SKCM: skin cutaneous melanoma, STAD: stomach adenocarcinoma, TGCT: testicular germ cell tumors, THCA: thyroid carcinoma, THYM: thymoma, UCEC: uterine corpus endometrial carcinoma, UCS: uterine carcinosarcoma, UVM: uveal melanoma. ** *p* < 0.01, *** *p* < 0.001, Asterisks were normal compared with tumor. (B) The survival curve of PTPN11 in multiple cancers.
