# Supplementary material for: Mometasone Furoate Inhibits the Progression of Head and Neck Squamous Cell Carcinoma via Regulating Protein Tyrosine Phosphatase Non-Receptor Type 11
Source: Biomedicines. 2023 Sep 22;11(10):2597. doi: 10.3390/biomedicines11102597 (PMC10603855; doi:10.3390/biomedicines11102597)
Supplement: Supplementary file 1 [file biomedicines-11-02597-s001.zip › biomedicines-2527257-supplementary.pdf]

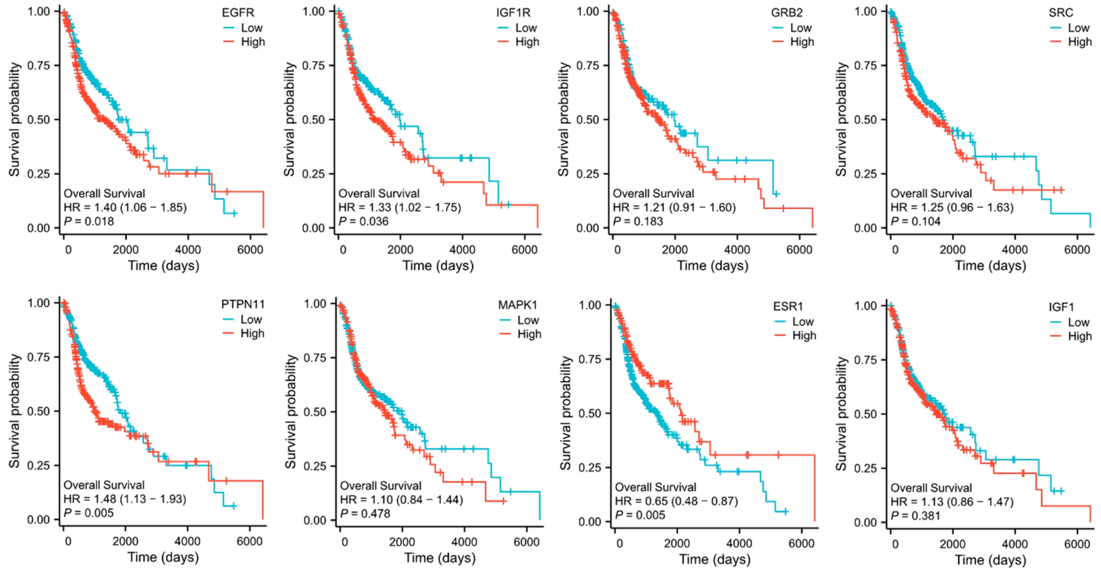

Supplementary Figure S1：Survival curve of patients with HNSCC in TCGA database.

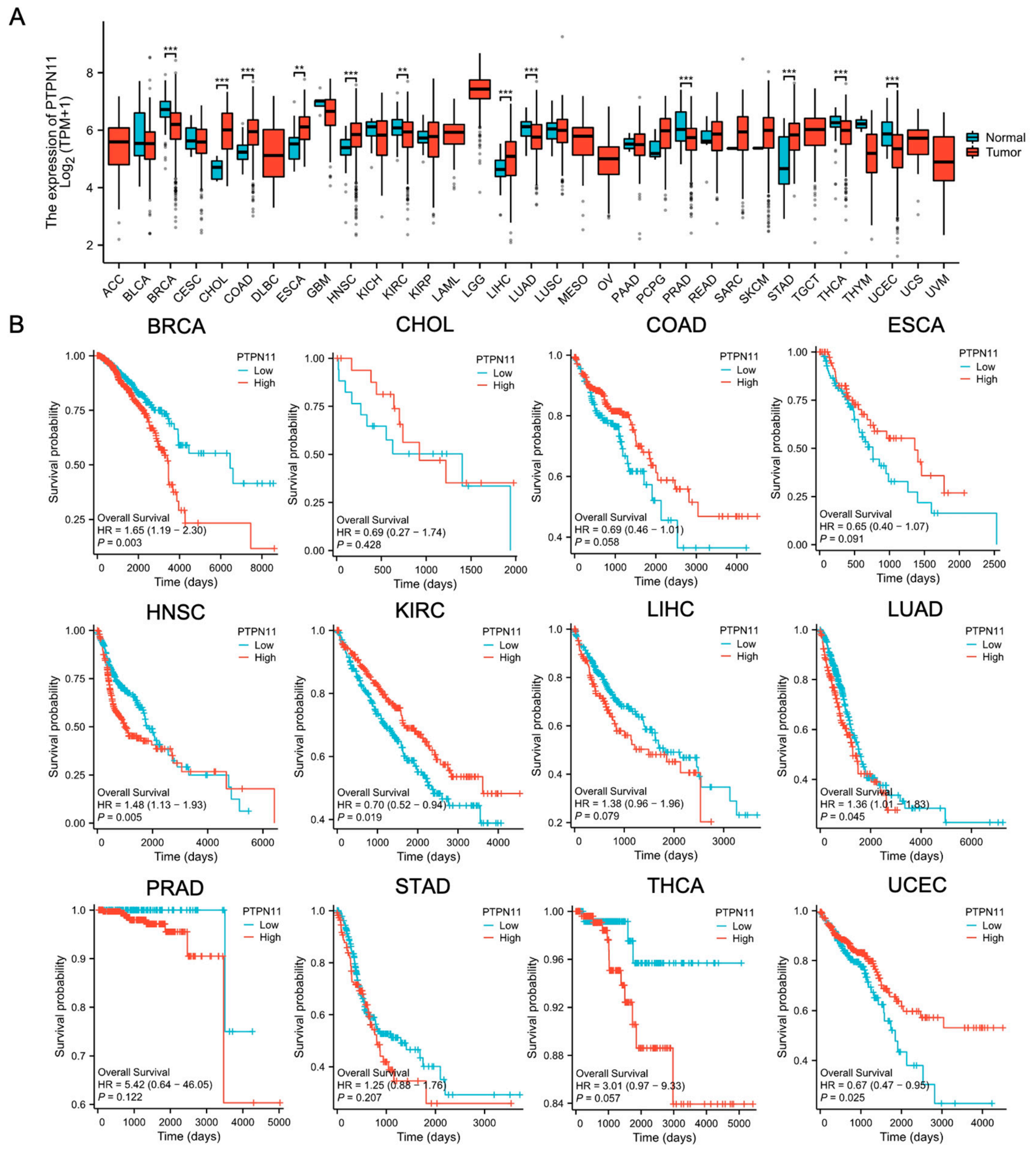

Supplementary Figure S2：Bioinformatics analysis of PTPN11 in multiple cancers in the TCGA database. A: The expression characteristics of PTPN11 in multiple cancers. ACC: Adrenocortical carcinoma, BLCA: Bladder urothelial carcinoma, BRCA: Breast invasive carcinoma, CESC: Cervical endocervical adenocarcinoma and squamous cell carcinoma, CHOL: Cholangiocarcinoma, COAD: Colon adenocarcinoma, DLBC: Lymphoid neoplasm diffuse large b-cell lymphoma, ESCA: Esophageal carcinoma, GBM: Glioblastoma multiforme, HNSC: Head and neck squamous cell carcinoma, KICH: Kidney chromophobe, KIRC: Kidney renal clear cell carcinoma, KIRP: Kidney renal papillary cell carcinoma, LAML: Acute myeloid leukemia, LGG: Brain lower grade glioma, LIHC: Liver hepatocellular carcinoma, LUAD: Lung adenocarcinoma, LUSC: Lung squamous cell carcinoma, MESO: Mesothelioma, OV: Ovarian serous cystadenocarcinoma, PAAD: Pancreatic adenocarcinoma, PCPG: Pheochromocytoma and paraganglioma, PRAD: Prostate adenocarcinoma, READ: Rectum adenocarcinoma, SARC: Sarcoma, SKCM: Skin cutaneous melanoma, STAD: Stomach adenocarcinoma, TGCT: Testicular germ cell tumors, THCA: Thyroid carcinoma, THYM: Thymoma, UCEC: Uterine corpus endometrial carcinoma, UCS: Uterine carcinosarcoma, UVM: Uveal melanoma. ** $P < 0.01$, *** $P < 0.001$, * was normal compared with tumor. B: The survival curve of PTPN11 in multiple cancers.
